# Mercury Production and Use in Colonial Andean Silver Production: Emissions and Health Implications

**DOI:** 10.1289/ehp.1104192

**Published:** 2012-02-14

**Authors:** Nicholas A. Robins, Nicole A. Hagan

**Affiliations:** 1 Department of History, North Carolina State University, Raleigh, North Carolina, USA; 2 Oak Ridge Institute for Science and Education Fellow, U.S. Environmental Protection Agency, National Center for Environmental Assessment, Research Triangle Park, North Carolina, USA

**Keywords:** health effects, Huancavelica, Peru, mercury emissions, mercury production, Potosí, Bolivia, silver production

## Abstract

Background: Colonial cinnabar mining and refining began in Huancavelica, Peru, in 1564. With a local source of mercury, the amalgamation process was adopted to refine silver in Potosí, Bolivia, in the early 1570s. As a result, large quantities of mercury were released into the environment.

Objectives: We used archival, primary, and secondary sources to develop the first estimate of mercury emissions from cinnabar refining in Huancavelica and to revise previous estimates of emissions from silver refining in Potosí during the colonial period (1564–1810).

Discussion: Although other estimates of historical mercury emissions have recognized Potosí as a significant source, Huancavelica has been overlooked. In addition, previous estimates of mercury emissions from silver refining under-estimated emissions because of unrecorded (contra-band) production and volatilization of mercury during processing and recovery. Archival descriptions document behavioral and health issues during the colonial period that are consistent with known effects of mercury intoxication.

Conclusions: According to our calculations, between 1564 and 1810, an estimated 17,000 metric tons of mercury vapor were emitted from cinnabar smelting in Huancavelica, and an estimated 39,000 metric tons were released as vapor during silver refining operations in Potosí. Huancavelica and Potosí combined contributed > 25% of the 196,000 metric tons of mercury vapor emissions in all of Latin America between 1500 and 1800. The historical record is laden with evidence of mercury intoxication consistent with effects recognized today. Our estimates serve as the foundation of investigations of present-day contamination in Huancavelica and Potosí resulting from historical emissions of mercury.

Widespread mercury pollution is not a -modern phenomenon. Archival documents and historical accounts can provide a perspective on mercury pollution and intoxication over several centuries. This historical perspective is important for current-day estimates of human exposure to anthropogenic versus natural sources of mercury and for estimating the accumulation of mercury pollution in the environment over time. Although large-scale mercury production and use have ceased, some of the mercury historically used in the Andes (South America) may potentially remain bioavailable and pose a risk to the present population. In addition, historical accounts provide documentary evidence that the adverse effects of mercury were already known and suggest that mercury exposure was of importance for certain historical events.

The hope of finding gold attracted many Spanish conquistadores to the Americas, but it was silver that they found in abundance. Central to silver mining and refining was the use of the mercury amalgamation technique and forced Indian labor. These factors were especially pronounced in the Andean region, resulting in widespread health effects in mining towns and the decimation of native communities through mining-related deaths and flight from the labor draft (Bargallo 1969; Cole 1985; Crespo Rojas 1970; Evans 1981; Liñan y Cisneros 1859; Salinas y Cordoba 1957).

Between 1550 and 1800, miners and refiners produced approximately 136,000 metric tons of silver in Latin America, or about 80% of global production during this time, releasing up to 196,000 metric tons of mercury as a result of amalgamation (Nriagu 1993). Globally, from 1550 to 1930, > 236,000 metric tons of mercury vapor were released from silver and gold refining using mercury amalgamation (Anonymous 1951; Crespo Rojas 1970; Cross 1983; Flynn and Giraldez 1996; Lacerda 1997; Marichal 2006; Nriagu 1993, 1994). The affinity between mercury and gold and silver was noted during Roman times and described in 1540 (Agricola 1950; Nriagu 1994), and the technique of mercury amalgamation, which enables the refining of lesser quality ores than is possible with smelting, first came into industrial use for silver refining in New Spain (present-day Mexico).

By the early 1570s, the mercury amalgama-tion process had been adopted in the Andes, enabling sustained, industrial-scale refining of abundant lesser-quality ores unsuitable for smelting. Andean silver miners obtained most of their mercury from the mine in the Santa Barbara Hill, just outside of Huancavelica, Peru, for most of the colonial period. Reflecting the importance of quicksilver to the Spanish Crown, in the early seventeenth century the Peruvian viceroy Luis de Velasco succinctly noted that “if there was not mercury, nor would there be silver” (de Velasco 1921). Other contemporaries would describe Huancavelica as “the soul of all of the mining towns in the Kingdom” that brought “prosperity and wealth to the” realm (Bueno 1951; Solórzano y Pereyra 1972). The introduction of the amalgamation process, coupled with the construction of lagoons and hydraulic mills and the imposition of draft labor in the mines and mills, led to the resurgence of the economy of Potosí, Bolivia.

Mining and refining operations in Huancavelica and Potosí depended on a draft labor system known as the *mita* to ensure sufficient workers. Men between 18 and 50 years of age from the areas surrounding Huancavelica and Potosí were subject to this levy and were required to work 2 months in Huancavelica and 12 months in Potosí. The *mita* system played a central role in the destruction of indigenous communities—not only through death and morbidity as a result of working in the mines and mills but also through the migration of people away from their home towns to evade the levy, which many considered a death sentence (Robins 2011).

Using archival, primary, and secondary sources, we developed an estimate of historical mercury emissions from cinnabar refining in Huancavelica and silver refining in Potosí. We reviewed historical information on processes and evidence of human exposures and health effects and examined uncertainties in estimating emissions. Our estimates serve as the foundation of community-wide and residential investigations in other studies of present-day mercury contamination in Huancavelica and Potosí resulting from historical emissions of mercury (Hagan et al. 2010).

## Mercury Production, Human Exposure, and Heath Effects in Huancavelica

*Mercury mining.* In Huancavelica, laborers extracted the cinnabar ore from the Santa Barbara Hill and transported it to smelters that surrounded the city. After preparing and heating the ore, the mercury it contained was vaporized, condensed, collected, stored, and sent to mining centers, including Potosí. In 1604, an advocate for the natives, Damían de Jeria, described how the vapors, dust, and candle smoke in the mine led to a “cough and a certain illness . . . called the illness of Huancavelica” (Sala Catala 1987) and asserted that to force people to work in the mercury mines was to send them “to the slaughterhouse” (Brown 2001). In 1630, another contemporary described the mines as “a living image of death, and a black shadow of hell” (Salinas y Cordoba 1957).

*Mercury refining.* Although fewer people were involved in refining mercury than extracting cinnabar ore, they were exposed to greater concentrations of mercury vapor as a result of the primitive manner in which the cinnabar smelters were constructed. Writing of the refining process around 1590, the Jesuit José de Acosta described how “if some smoke or vapor comes to the people who open the pots, they get mercury poisoning and die, or remain in a very bad state or lose their teeth” (de Acosta 1987). In the nineteenth century, an anonymous writer described the poor construction of and the ease with which mercury escaped from the smelters (Anonymous 1857).

Exacerbating the situation, the smelters were commonly opened before they had cooled to reduce the refining time, which could lead to acute exposures to high mercury concentrations (Anonymous 1857; Menéndez Navarro 1996). It was not only male workers who were poisoned; their families often helped prepare, operate, and clean the ovens. In addition, mercury readily crosses the placental barrier, thus putting infants at risk of *in utero* exposure and subsequent physical and mental develop-mental abnormalities if they survived pregnancy (Agency for Toxic Substances and Disease Registry 1999; Alcser et al. 1989; Anonymous 1857; Barlow et al. 1994; Clarkson and Magos 2006; Evans 1998; Menéndez Navarro 1996; Rowland et al. 1994; U.S. Environmental Protection Agency 1997; Yeates and Mortensen 1994).

Written historical accounts also noted the links between air pollution from mercury smelting and illness. Antonio de Ulloa, the governor of Huancavelica from 1758 to 1764, believed that thermal inversions were among the possible causes of childhood respiratory illnesses in the town, which he believed was partially caused by “the sulphourous smoke that they continually breathe, coming from the ovens in which they extract the mercury, which are in such abundance, that in summer time with the freezes, form a dense cloud, that covers the area of the town” (de Ulloa 1992).

So dreaded—and lethal—was work in the Santa Barbara mine and smelters of Huancavelica that by the late 1500s the surrounding communities that provided draft labor were for the most part abandoned. Although many died from mercury intoxication in Huancavelica and others returned home permanently incapacitated, still others fled to districts that were exempt from the labor levies or to areas that were free of Spanish domination (Fernández de Castro y Andrade 1951; Patiño Paúl Ortíz 2001; Salinas y Cordoba 1957).

## Silver Production, Human Exposure, and Heath Effects in Potosí

Potosí city was the largest city in the world in 1650 (population 160,000), and many more people were exposed to mercury vapor as a consequence of silver refining in Potosí than from cinnabar mining and refining in Huancavelica, where the population never exceeded 15,000 (Arena 1901; Bakewell 1975; Brown 1988; Caravantes 1989; Lohmann Villena 1999; Patiño Paúl Ortíz 2001; Roel Pineda 1970; Whitaker 1941). Like Huancavelica, Potosí depended on draft labor for the least desirable and most dangerous aspects of mining and refining.

Although accidents were more common in the silver mines, workers in the silver refining mills suffered greater exposure to mercury both in liquid and vapor forms. Most of the mills in Potosí were hydraulic, powered by water from lagoons above the city, and many were centrally located, some being only a few blocks from the city’s main plaza (Cobb 1977; Cobo 1956; Cole 1985). Consequently, exposure to mercury was not limited to occupational settings.

After silver ore was ground to the consistency of flour, it was mixed with water, salt, mercury, iron, and other ingredients and spread out on a stone patio. Because of the scarcity and cost of fuel, forced laborers would tread upon it for about a month, usually barefoot and up to their knees, to facilitate and accelerate the amalgamation process [Archivo y Biblioteca Nacionales de Bolivia, Audiencia de la Plata (ABNB ALP) Minas 15/1 1556].

When the amalgamation process was complete, the paste was washed to separate the amalgam by flowing water over it in a trough. Runoff was generally recaptured and repeatedly reprocessed to maximize extraction of the mercury and silver. As a result, approximately 85% of the mercury brought to Potosí was ultimately released as a vapor into the atmosphere, with the remainder being released into local waterways. Once separated, the amalgam was squeezed in a cloth tube to further separate the mercury from the silver before it was placed in a conical mold and fired, releasing mercury vapor into the atmosphere (Capoche 1959; Caravantes 1989; de Acosta 1987; de Murua 1987; Fuentes Bajo 1986; Lohmann Villena 1999; Mira Delli-Zotti 1988).

Despite their partial knowledge of the effects of mercury poisoning, written historical accounts remarked on its toxic nature in silver production. In 1629, the priest Pedro de Oñate (1951) observed how

[W]e well know and have seen . . . how terrible are the effects of mercury, as only in smelting . . . and treading . . . many are poisoned by mercury and we see those effects among those to whom we give last rites.

Similarly, Bartolomé Arzáns de Orsúa y Vela, a chronicler of Potosí writing in the early eighteenth century, made frequent references to birth deformities, stillbirths, and mental illness (Arzáns de Orsúa y Vela 1965). Although such references are not proof of mercury intoxication, they do illustrate conditions in the city.

Similarly, Potosí’s reputation for aggressive behavior does not directly indicate widespread mercury intoxication but is consistent with it. Indeed, the city was frequently beset with paroxysms of violence, often expressed along ethnic and class lines (Arzáns de Orsúa y Vela 1965; Baquijano 1793; Cole 1985; Cook 1981; Crespo Rojas 1970; Helmer 1960; Martínez y Vela 1939; Montesinos 1906). Outbreaks of violence in 1593 and 1600 presaged a bloody ethnic conflict that consumed Potosí from 1622 to 1624, resulting in the deaths of > 5,000 people (Arzáns de Orsúa y Vela 1965; Baquijano 1793; Cook 1981; Crespo Rojas 1970; Helmer 1960; Martínez y Vela 1939).

Violence in Potosí was a prominent theme for Arzáns de Orsúa y Vela, which he charac-terized as an “irremediable plague,” “fatal habit,” and “a custom of the land” (Martínez y Vela 1939). The cleric Antonio de la Calancha (1974) pondered,

Is there a town in the world like Potosí where there are so many quarrels, and such routine killings, even between the best of friends . . . [and] where against the Indians are seen so many cruelties by the covetous?

The answer to Friar de la Calancha’s query may literally have been in the air the residents breathed. While some contemporaries noted the insalubrious nature of the smoke, others linked the prevalence of violence in the city to the environment (Arzáns de Orsúa y Vela 1965; de Matienzo 1918). For example, in 1759, an anonymous writer described Potosí, with the

thick cloud that forms . . . over the city, and is clearly seen on any clear moonlit night, this is without doubt . . . vapors and poisonous fumes . . . from dead animals, from trash heaps, and other fine dust from the ore and from the mercury smoke in the burning and reburning of the [silver. This] . . . mix of bad vapors and fumes cannot be healthful. (Anonymous 1759)

Although smelting of the silver ores also may have released lead compounds that could have contributed to health effects, including effects on behavior, the quantity emitted would have been significantly less than the amount of mercury added to the ore for the amalgamation process. Thus, limited evidence suggests that it was not a major contributor to historical health effects.

## Estimating Historical Mercury Emissions

Estimating mercury emissions in Huancavelica and Potosí is complicated by several factors, including uncertainties concerning production records and the efficiency of mercury smelters and the silver refining process. The amount of mercury lost in both processes varied according to the skill of the refiner, the integrity of the seals in the smelters, and—in the case of silver refining—the grade of the ore, with richer ores consuming more mercury than poorer ores. The amount of unregistered production, or contraband, is also uncertain and is likely to have increased during periods of declining production as refiners sought to avoid taxation (Bakewell 1975; Brading and Cross 1972; Capoche 1959; Cooke et al. 2009; Lohmann Villena 1999).

Unregistered production clearly accounted for a significant proportion of the total mercury and silver produced (ABNB ALP Minas 63/17 1655; Bakewell 1975; Caravantes 1989; Cobb 1977; Cobo 1956; Molina Martínez 1995; Ramírez 1906; Whitaker 1941). Estimates of unrecorded mercury production range from 10% to 66% of the amount of recorded production, and estimates of contra-band silver production range from 20% to 66% of the amount of recorded production, depending on the time period. Based on a review of the evidence, it appears that the average rate was 25–30% (ABNB ALP Minas 112/8 1587; Arena 1901; Bakewell 1975; Brown 1988; Cañete y Domínguez 1952; Cobb 1977; Cole 1985; Cook 1957; Cross 1983; de Acosta 1987; de la Calancha 1974; Fisher 1977; Roel Pineda 1970; Salinas y Cordoba 1957; Válcarcel 1957; Whitaker 1941).

*Estimated mercury emissions in Huancavelica.* We examined colonial records of mercury production in Huancavelica from multiple sources (Arena 1901; Brown 1988; Caravantes 1989; Fisher 1977; Lohmann Villena 1999; Patiño Paúl Ortíz 2001; Whitaker 1941). Based on these records, we estimate that from 1570 to 1810, 50,600–51,300 metric tons of mercury were produced in Huancavelica, not including contraband. Assuming that approximately 25% of the mercury produced in Huancavelica was not reported to the government (contraband), we estimate the total amount of mercury produced in Huancavelica during this time was approximately 68,200 metric tons.

Historical estimates of emissions during mercury smelting generally ranged from 10% to 50% of the mercury used. Based on an examination of various sources, a factor of 25% appears reasonable (Anonymous 1857; Arena 1901). This is corroborated by a study in 2008 of 22 Chinese artisan smelters where emissions ranged from approximately 7% to 32%, with a mean of approximately 20% (Li et al. 2008). Applying an emissions factor of 25%, we estimate that the total amount of mercury vapor released between 1564 and 1810 from cinnabar refining in Huancavelica was approximately 17,000 metric tons, an annual average of 69 metric tons of mercury vapor.

*Estimated mercury emissions in Potosí.* Mercury loss occurred at several stages of silver processing. The ratio of mercury consumed per pound of silver produced was a function of the quality of the ore and skill of the refiners, both of which varied over time, resulting in estimates ranging from 1:1 to 2:1 [Bakewell 1975; Casa Nacional de Moneda, Archivo Histórico (CNMAH) CR 554 1705; CNMAH CR 620 1717; CNMAH CR 651/427 1720; CNMAH CR 721 1734; CNMAH CR 810/238 1764]. Although mercury could volatize during the treading process, this appears to have been quite limited because it was generally trapped in the ore mixture. More mercury was lost in the washing process, with some contemporaries estimating a loss of 10%. Amalgam recovered after washing was dried before being smelted to recapture the mercury and silver, which was added back to new batches of ore. Most of the mercury loss is believed to have occurred during the final step of refining, when the amalgam was placed in a retort and heated. Although some of the mercury was recaptured and reused, the mercury that was not lost in treading or runoff from washing was ultimately volatized (ABNB ALP Minas 112/8 1587; Arena 1901; Cañete y Domínguez 1952; Cobb 1977; Cole 1985; Cook 1957; de Acosta 1987; de la Calancha 1974; Purser 1971; Roel Pineda 1970; Salinas y Cordoba 1957; Válcarcel 1957). Recognizing the prevalence of the reprocessing of runoff is critical because it increases overall emissions relative to emissions that would result from a single firing of amalgam. It also places emissions at the upper limit of recent estimates of emissions factors from artisanal gold mining, which range from 30% to 83% (Strode et al. 2009). Therefore, to estimate historical emissions, we assumed a conversion ratio of 1.7 pounds of mercury per pound of silver produced, which incorporates an estimated loss of 15% of the mercury via treading and runoff, a conservative estimate (de Murua 1987; Fuentes Bajo 1986; Mira Delli-Zotti 1988; Nriagu 1993, 1994).

Detailed information on the derivation of our estimates for silver production and mercury use in Potosí city is provided in Supplemental Material (http://dx.doi.org/10.1289/ehp.1104192). In brief, based on detailed registries of annual silver production in Potosí district for 1574–1735 and Potosí city for 1660–1720 (Bakewell 1975), and assuming an additional 25% of the total was unrecorded (contraband), we estimated that 18,000 metric tons of silver were produced in Potosí city from 1574 to 1735. Multiplying the total by 1.7 pounds of mercury consumed per pound of silver produced yields an estimated average of 190 metric tons of mercury consumed in the city of Potosí per year, or 30,600 metric tons of mercury over the 162-year period (1574–1735) (Bakewell 1975).

We estimated that 1,600 metric tons of silver were produced in Potosí city from 1736 through 1760 based on detailed records of silver production in Potosí district documented by Cross (1983), resulting in the consumption of approximately 2,700 metric tons of mercury, or 108 metric tons per year. Because records were not available from 1760 to 1810, we estimated production using the average annual values from 1735 to 1760, a period of relatively low silver production and mercury consumption. This resulted in an estimate of 39,000 metric tons of mercury consumed in Potosí city from 1574 to 1810, or 165 metric tons of mercury per year.

## Conclusions

The introduction of the mercury amalgamation process to refine silver in Latin America played a vital role in the development of the modern global economy (Robins 2011). It also resulted in the poisoning and death of countless people, including workers and other residents of Huancavelica, Potosí, and other mining centers. Based on an analysis of mercury production in Huancavelica and its consumption in Potosí, and taking into account contraband production and inefficiencies in the refining process, we estimated that approximately 17,000 metric tons of mercury vapor were released into the atmosphere in Huancavelica between 1564 and 1810, and that 39,000 metric tons of mercury vapor were released in Potosí between 1574 and 1810.

Despite the limited knowledge of contemporaries concerning the effects of mercury, the historical record is laden with descriptions of physical and psychological impairments that would be consistent with the known effects of mercury. The impacts of cinnabar refining and silver smelting not only took a toll on the health of colonial residents, but remain in Huancavelica and Potosí, still haunting the current residents today. As we have demonstrated in field research, widespread mercury contamination is still present in both towns, in ambient soil and in residences constructed of adobe brick (Hagan et al. 2010). Therefore, in addition to providing a historical perspective on the extent and consequences of mercury exposures resulting from colonial cinnabar and silver mining in Huancavelica and Potosí, our estimates of past emissions will continue to guide current and future research in these towns.

## Supplemental Material

(57 KB) PDF
