# Supplementary material for: Mercury Production and Use in Colonial Andean Silver Production: Emissions and Health Implications
Source: Environ Health Perspect. 2012 Feb 14;120(5):627–31. doi: 10.1289/ehp.1104192 (PMC3346781; doi:10.1289/ehp.1104192)
Supplement: (57 KB) PDF [file ehp.1104192.s001.pdf]

Supplemental Material

Title:
Mercury Production and Use in Colonial Andean Silver Production: Emissions and Health Implications

Authors:
Nicholas A. Robins[1,2] and Nicole A. Hagan[2]

Affiliations:
[1] Department of History, North Carolina State University, Raleigh, NC, 27695
[2] Oak Ridge Institute for Science and Education Fellow, United States Environmental Protection Agency, National Center for Environmental Assessment, Research Triangle Park, NC, 27711

Estimated Mercury Emissions in Potosí

Bakewell (1975) provides a detailed registry of annual silver production in Potosí district from 1574 to 1735. Silver production is reported in units of marcos, a Spanish unit used during the colonial period. The conversion factors used in the calculations are shown in Supplemental Material, Table 1. The total amount of silver production reported for Potosí district from 1574 to 1735 is equal to 73,351,672.00 marcos, or 16,874.19 tons.

Supplemental Material, Table 1. Unit conversions

2 marcos	=	1 Spanish pound
1 Spanish pound	=	460.09 grams
1 peso	=	1 gram
1,000,000 grams	=	1 metric ton

Potosí district is the entire area encompassed by "Potosí" and contains the city of Potosí. While silver refining occurred throughout the entire district, the predominant area of silver production was in the city of Potosí. Bakewell (1975) also provides a detailed registry of annual silver production in Potosí city from 1660 to 1720. The percentage of silver produced in Potosí city was determined by dividing the amount of silver produced in Potosí city by the amount of the total amount of silver produced in Potosí district. The percentage of silver production

attributable to the city was averaged across 1660 through 1720, resulting in 80.12% of the total silver production being produced in Potosí city.

The total amount of silver reported in Bakewell (1975) needed to be adjusted for contraband. Approximately 25% of all silver produced in Potosí district went unreported to the government in order to avoid taxation, and therefore is not included in Bakewell's (1975) records. The total amount of silver produced in Potosí district including contraband is 22,498.91 tons. Of this adjusted total, 80% was produced in Potosí city, or 17,999.13 tons.

The conversion ratio of 1.7 pounds of mercury per pound of silver produced reflects a conservatively estimated 15% that was likely lost in treading and runoff (Fuentes Bajo 1986; Mira Delli-Zotti 1988; Murua 1987; Nriagu 1993, 1994). Estimates of conversion rates vary, and were a function of the quality of the ore and skill of the refiners, both of which varied over time and could range anywhere from 1:1 to 2:1 (Bakewell 1975; CNMAH CR 554 1705; CNMAH CR 620 1717; CNMAH CR 651/427 1720; CNMAH CR 721 1734; CNMAH CR 810/238 1764). Multiplying the total amount of silver produced in Potosí city by 1.7 results in 30,598.52 tons of mercury being emitted to the atmosphere between 1574 and 1735.

The amount of mercury released into the atmosphere during the later part of the colonial period (1735-1810) used detailed records of silver production from Potosí district documented by Cross (1983). The total production from 1736-1760 is reported for five year periods. The amount of total silver produced for each of these five year periods was converted into the amount of mercury emitted in Potosí city as described above. The resulting numbers are shown in

Supplemental Material, Table 2. From 1736 to 1760, 1582.72 tons of silver were produced in Potosí city, resulting in the release of 2690.63 tons of mercury vapor. This is an annual average of 112.11 tons of mercury per year.

Supplemental Material, Table 2. Mercury emissions from 1736-1760

Years	Total Ag production in Potosí district (pesos)	Total Ag production in Potosí district (tons)	Total Ag production adjusted for contraband (tons)	Total Ag production in Potosí city (tons)	Total Hg emissions in Potosí city (tons)
1736-1740	8566766.00	246.29	328.39	262.71	446.61
1741-1745	8267747.00	237.70	319.93	253.54	431.03
1746-1750	10082220.00	289.86	386.49	309.19	525.62
1751-1755	11765544.00	338.26	451.01	360.81	613.38
1756-1760	12928272.00	371.69	495.58	396.47	673.99

Because records were not available for 1760 to 1810, the latter part of the colonial period of interest, the amount of mercury emissions were extrapolated using the annual average mercury emissions derived for 1735 to 1760 from Cross (1983). The annual average of 112.11 tons of mercury emitted per year was multiplied by 50 years, resulting in 5605.48 tons of mercury emitted between 1760 and 1810.

By summing the total mercury emissions from the three time periods, the total amount of mercury emitted from the amalgamation-based silver production process equals 38,894.63 tons, or an average of 164.81 tons per year.

References

Bakewell P. 1975. Registered Silver Production in the Potosí District, 1550-1735. Jahrbuch fur Geschichte von Staat, Wirtschaft und Gesellschaft Lateinamerikas 12: 67-103.

CNMAH, CR 554. 1705. Libro borrador en que se asientan las partidas del azogue que se distribuye entre los azogueros y mineros de esta ribera y minerales de su contorno desde veinte y tres de mayo de este año de mil setecientos y cinco en adelante. 1705-1717: 17-131.

CNMAH, CR 620. 1717. Libro o borrador en que se asientan las partidas del azogue que se distribuye de los reales alamacenes de esta caja, entre los azogueros y mineros de la rivera de esta Villa y minerales de su contorno, que corre desde cinco de mayo de este año de mil setecientos y diez y siete en adelante. Potosí, 1717-1734: 13-196.

CNMAH, CR 651/427. 1720. Libro del cargo y data de los azogues que entran y salen de los reales almacenes de la real caja de la Villa Imperial de Potosí. Potosí, 1720-1740.

CNMAH, CR 721. 1734. Libro borrador en que se toma razón de las partidas del azogue que se distribuye de los reales almacenes de esta caja, entre los azogueros y mineros de la rivera de esta Villa de Potosí y otras partes. Potosí, 1734-1746: 9-81

CNMAH, CR 810/238. 1964. Libro real donde se anota la distribución de azogues. Potosí, 1764-1773.

Cross H. 1983. South American bullion production and export 1550-1750. In: Precious Metals in the Later Medieval and Early Modern Worlds. Richards JF, ed. Durham, NC: Carolina Academic Press.

Fuentes Bajo MD. 1986. El azogue en las postrimerías del Perú colonial [in Spanish]. Revista de Indias 46:75-105.

Mira Delli-Zotti G. 1988. Reformas bourbonicas en America. La provision de azogue en el Virreinato del Rio de la Plata [in Spanish]. Cuadernos Hispanoamericanos 2: 209-222.

Murua M de. 1987. Historia general del Perú [in Spanish]. Ballesteros Gaibrois M, ed. Madrid: Historia 16.

Nriagu J. 1993. Legacy of mercury pollution. Nature 363:6430:589.

Nriagu J. 1994. Mercury pollution from the past mining of gold and silver in the Americas. Sci Total Environ 149:167-181.
